# Unpacking the dangers of super absorbent polymer water beads: an *in vitro* analysis

**DOI:** 10.3389/fped.2025.1477506

**Published:** 2025-03-12

**Authors:** Jack J. Hachem, Javier Monagas, Ankona Banerjee, Robert A. Noel

**Affiliations:** ^1^ Department of Pediatric Gastroenterology, Baylor College of Medicine, Children’s Hospital of San Antonio, San Antonio, TX, United States; ^2^ Department of Pediatric Gastroenterology, Baylor College of Medicine, Baylor College of Medicine, Houston, TX, United States

**Keywords:** pediatric bowel obstruction, foreign body ingestions, super absorbent polymer beads, water beads, polyethylene glycol 3350

## Abstract

**Objectives:**

This study aims to investigate the risk of pediatric bowel obstruction from various types of super absorbent polymer (SAP) beads in different liquid media, explore treatment options, and develop a clinical decision-making algorithm for healthcare providers.

**Methods:**

Three experiments were conducted involving the measurement of SAP beads' expansion in different liquid media. The first experiment examined the expansion of beads in tap water, gastric fluid, and small intestine fluid. The second compared the expansion of beads from six manufacturers in water. The third tested the effect of varying concentrations of Polyethylene Glycol 3350 on bead expansion.

**Results:**

The study found that SAP beads reached their largest size in water, with significant size-dependent and solution-specific effects on their expansion. Large beads had a *β* of 12.67 (95% CI: 10.25–15.1; *p* < 0.001) compared to small beads. Gastric acid reduced expansion with a *β* of −7.01 (95% CI: −9.67 to 4.35; *p* < 0.001) and alkaline solution with a *β* of −3.88 (95% CI: −6.54 to 1.23; *p* = 0.002) compared to water. Treatment solutions containing high concentrations of polyethylene glycol (PEG) 3350 effectively reduced the size of the beads (*p* < 0.001).

**Conclusions:**

This research highlights the importance of understanding the characteristics and risks associated with SAP beads to mitigate the dangers they pose to pediatric populations. Our findings underscore the need for standardized management of SAP bead ingestion, which could improve patient outcomes while reducing unnecessary diagnostic and therapeutic interventions. Further research and clinical validation of these strategies *in vivo* are essential to develop safe and efficient protocols for managing SAP bead ingestions

## Introduction

The *Pediatrics Journal* cites an incidence rate of 17.9 foreign body ingestions per 10,000 children under six years old in the United States (US) (1). The demographic most frequently affected by these incidents includes children aged one year (21.3%) and boys (52.9%), with toys accounting for nearly 10% of cases. Super absorbent polymer (SAP) beads are particularly concerning, as they have been associated with bowel obstruction in several case studies (2). Initially created in the 1960s by the US Department of Agriculture for soil water conservation, SAPs have since permeated mainstream toy production. When ingested, these beads can continue to expand by absorbing water in the small intestine, potentially causing bowel obstruction in the duodenal sweep or ileocecum. Recent cases of intestinal obstruction tied to SAPs have sparked serious concerns, prompting major retailers to remove such products from their shelves (3). However, due to their commercial appeal, similar beads continue to be marketed to adults as decorative items. From 2007 to 2022, there were over 8,000 estimated visits to US emergency departments linked to water beads, with these visits more than doubling in the last 2 years of the study. This increase occurred despite product recalls and existing voluntary safety standards, indicating that current prevention strategies are insufficient (4). Further emphasizing the rising problem, a study using the National Electronic Injury Surveillance System (NEISS) identified 226 water bead injuries from 2013 to 2023, with 66% involving ingestion. Children under age 2 years accounted for 29% of injuries, and multiple water beads were involved in 56% of cases. A significant uptrend in injury frequency was observed after 2020, with 7% of cases requiring escalation of care. These findings highlight the increasing prevalence and significant harm associated with water bead injuries, particularly affecting children under 5 years of age (5).

This study aims to investigate the potential risk of pediatric bowel obstruction posed by different types of SAP beads in various liquid media. We also seek to explore possible treatments and develop a clinical decision-making algorithm to aid healthcare providers when dealing with cases of children ingesting these polymers. The urgency of this research is emphasized by the significant adverse outcomes that can result from improper management of these ingestions. Establishing a standardized, evidence-based approach to handling ingested superabsorbent polymers could prove to be an invaluable tool for clinicians. By potentially decreasing the morbidity and mortality rates associated with these ingestions, this research underscores the importance and necessity of understanding and addressing this issue.

## Materials and methods

This study was organized around three meticulously planned experiments. The diameters of the super absorbent polymers were measured in millimeters using an Esydon® Digital Caliper (ASIN: B0C86NSBDK).

The first experiment aimed to determine the effect of three different control solutions: tap water, simulated gastric fluid, and simulated small intestine fluid, on the expansion of water beads. The simulated small intestine fluid was prepared by buffering 180 ml of lactated ringer fluid with an osmolality of 265 mOsm/kg to achieve a pH of 7.5–8.0. The simulated gastric fluid was purchased from Carolina Biologics Company®, composed of water (99%), pepsin (0.5%), hydrogen chloride (0.22%), and Thymol (0.1%). With an approximate total molarity of HCl of 0.0603 M the pH of the 180 ml solution of simulated gastric fluid is approximately 1.22 and an osmolarity of 127.4 mOsm/kg. Super absorbent polymer beads were obtained from six major manufacturers: Leeche®, Ainolway®, Yiquo®, Babiya®, Waterballz®, and Orbeez®. The beads were categorized into small (<3 mm), medium (3–7 mm), and large (>7 mm) groups and measured in their dry state using an Esydon Digital Caliper®. Six beads from each group were submerged in 180 ml of each of the three control solutions and three beads out of those from each group were measured at 2, 6, 12, and 24 h. Three beads from each control solution were transferred to three different treatment solutions containing 17 grams of PEG 3350, 6 grams of psyllium fiber, and 10 grams of Saccharin, each mixed into 180 ml of water. These beads were then measured again after 24 h.

The second experiment involved a comparative study of water beads from six major manufacturers: Ainolway®, Cosmos®, and Magic Labs® (manufacturers of smaller beads), and Babiya®, Leeche®, and Yiquduo® (manufacturers of larger beads). All water beads were made from the same material, sodium polyacrylate gel. Ten beads from each manufacturer were measured and then immersed in 180 ml of water. Subsequent measurements were taken at intervals of 2, 12, and 24 h. The aim was to provide a direct comparison between the different manufacturers and improve measurement reliability.

The third experiment focused on the effect of varying concentrations of polyethylene glycol (PEG) 3350 on the maximum expansion size of large water beads (>7 mm). The beads were initially placed in 180 ml of simulated gastric juice, then transferred to 180 ml of alkaline fluid to simulate the pH change in the small intestine. Ten beads were immersed in each of the eight different PEG concentrations: 17 g, 34 g, 51 g, 68 g, and 85 g in 90 ml and 180 ml of water respectively. Measurements were taken at 0, 2, 12, and 24 h.

All analyses conducted were descriptive, employing summary statistics such as median and interquartile range for continuous variables, and frequency and proportion for categorical variables. Differences between groups were evaluated using nonparametric methodologies like the Kruskal–Wallis test for continuous variables and Fisher's exact test for categorical variables. Linear mixed modeling was utilized to assess changes in bead sizes over time and provide distribution estimates. Graphical representations of changes in bead sizes were shown using line and bar graphs. All analyses were performed using R software version 4.3.0 (R Core Team, Vienna, Austria), considering a *p*-value <0.05 as statistically significant.

## Results

### Experiment 1: effect of solution type on diameter over time

In the first experiment, water beads were categorized by size and exposed to different control solutions to observe their expansion over time. The expansion characteristics of super absorbent polymer beads were evaluated for small (<3 mm), medium (3–7 mm), and large (>7 mm) bead sizes across a range of control and treatment solutions. Measurements were taken at multiple time points (0, 2, 6, 12, and 24 h) to assess the dynamics of bead size changes under different conditions. Overall, the results revealed significant variation in the expansion potential of the beads depending on their size and the solution in which they were immersed.

The initial mean diameters prior to submersion were 1.97 mm, 5.48 mm, and 8.17 mm for small, medium, and large beads, respectively. At 24 h, the small beads expanded to a maximum size of 10.6 mm [3.43–10.6 mm], with a mean size of 8.35 mm (SD ± 2.79 mm). The medium beads reached a maximum size of 27.73 mm [14.7–27.7 mm], with a mean size of 21.8 mm (SD ± 5.28 mm). The large beads reached a maximum size of 32.4 mm [16.6–32.4 mm], with a mean size of 25.7 mm (SD ± 15.4 mm).

Small beads (<3 mm) expanded modestly in simulated gastric fluid, growing from 2.23 mm (SD = 0.225, range: 2.00–2.60 mm) to 3.43 mm (SD = 0.666, range: 3.00–4.20 mm) after 24 h. In contrast, they expanded significantly in tap water, reaching 10.6 mm (SD = 0.808, range: 9.70–11.3 mm) after 24 h. Simulated small intestine fluid also supported moderate growth to 7.00 mm (SD = 1.14, range: 6.20–8.30 mm) after 24 h. When transitioned to treatment solutions, PEG-treated beads expanded to 8.40 mm (SD = 1.61, range: 6.90–10.1 mm), while psyllium-treated beads grew further to 10.5 mm (SD = 1.47, range: 9.60–12.2 mm). Splenda-treated beads exhibited comparable growth, reaching 10.2 mm (SD = 0.513, range: 9.80–10.8 mm). Across all conditions, the overall mean size of small beads at 24 h was 8.36 mm (SD = 2.79, range: 3.00–12.2 mm).

Medium (3–7 mm) and large (>7 mm) beads exhibited greater expansion potential. Medium beads in tap water grew from 5.48 mm (SD = 0.223, range: 5.30–5.80 mm) to 27.7 mm (SD = 1.46, range: 26.2–29.1 mm) at 24 h, while those in simulated gastric fluid reached 14.7 mm (SD = 0.208, range: 14.5–14.9 mm). Simulated small intestine fluid facilitated significant expansion to 22.1 mm (SD = 0.265, range: 21.8–22.3 mm). For large beads, the most substantial growth occurred in tap water, where they expanded from 8.17 mm (SD = 0.758, range: 7.10–9.20 mm) to 32.4 mm (SD = 34.4, range: 9.50–72.0 mm). In simulated gastric fluid, large beads grew to 16.0 mm (SD = 5.93, range: 9.20–19.9 mm), and in simulated small intestine fluid, to 29.8 mm (SD = 17.8, range: 9.20–40.2 mm). Among the treatment solutions, saccharin-treated medium and large beads achieved the largest final diameters of 24.3 mm (SD = 2.30, range: 22.0–26.6 mm) and 31.4 mm (SD = 0.400, range: 31.0–31.8 mm), respectively. Overall, medium and large beads showed remarkable expansion across most conditions, particularly in tap water and saccharin solutions. See Table 1.

**Table 1:** Size-dependent effect on maximum growth.

Immersion fluid	Water				
Time when measured	0 h	2 h	6 h	12 h	24 h
	(*N* = 6)	(*N* = 3)	(*N* = 3)	(*N* = 3)	(*N* = 3)
Small beads: Size (mm)					
Mean (SD)	1.97 (0.463)	9.21 (0.788)	10.0 (0.947)	10.1 (0.925)	10.6 (0.808)
Median[Min, Max]	1.80 [1.70, 2.90]	8.93 [8.60, 10.1]	9.69 [9.30, 11.1]	9.72 [9.50, 11.2]	10.7 [9.70, 11.3]
Medium beads: Size (mm)					
Mean (SD)	5.48 (0.223)	15.4 (0.900)	18.0 (2.80)	24.0 (1.01)	27.7 (1.46)
Median[Min, Max]	5.40 [5.30, 5.80]	15.4 [14.5, 16.3]	18.9 [14.9, 20.3]	23.9 [23.1, 25.1]	27.9 [26.2, 29.1]
Large beads: Size (mm)					
Mean (SD)	8.17 (0.758)	19.9 (17.4)	26.3 (26.0)	29.3 (29.4)	32.4 (34.4)
Median[Min, Max]	8.30 [7.10, 9.20]	10.1 [9.50, 40.0]	13.1 [9.50, 56.2]	15.3 [9.50, 63.0]	15.7 [9.50, 72.0]
Immersion fluid	Gastric Acid				
Time when measured	0 h	2 h	6 h	12 h	24 h
	(*N* = 6)	(*N* = 3)	(*N* = 3)	(*N* = 3)	(*N* = 3)
Small beads: Size (mm)					
Mean (SD)	2.23 (0.225)	2.07 (0.0577)	3.47 (0.473)	3.30 (0.800)	3.43 (0.666)
Median[Min, Max]	2.15 [2.00, 2.60]	2.10 [2.00, 2.10]	3.30 [3.10, 4.00]	3.30 [2.50, 4.10]	3.10 [3.00, 4.20]
Medium beads: Size (mm)					
Mean (SD)	5.62 (0.264)	11.4 (2.21)	12.8 (2.65)	13.7 (2.12)	14.7 (0.208)
Median[Min, Max]	5.70 [5.20, 5.90]	10.4 [9.80, 13.9]	12.9 [10.1, 15.4]	14.7 [11.3, 15.2]	14.8 [14.5, 14.9]
Large beads: Size (mm)					
Mean (SD)	8.15 (0.589)	11.6 (3.52)	11.6 (3.59)	12.7 (3.55)	16.0 (5.93)
Median[Min, Max]	8.15 [7.40, 9.00]	9.85 [9.20, 15.6]	9.80 [9.20, 15.7]	12.6 [9.20, 16.3]	19.0 [9.20, 19.9]
Immersion fluid	LR (alkalotic)				
Time when measured	0 h	2 h	6 h	12 h	24 h
	(*N* = 6)	(*N* = 3)	(*N* = 3)	(*N* = 3)	(*N* = 3)
Small beads: Size (mm)					
Mean (SD)	2.15 (0.217)	6.35 (1.07)	6.65 (1.31)	7.05 (1.40)	7.00 (1.14)
Median[Min, Max]	2.10 [1.90, 2.50]	6.70 [5.14, 7.20]	7.30 [5.14, 7.50]	7.30 [5.54, 8.30]	6.50 [6.20, 8.30]
Medium beads: Size (mm)					
Mean (SD)	5.50 (0.228)	12.3 (0.854)	14.1 (1.82)	18.0 (3.35)	22.1 (0.265)
Median[Min, Max]	5.50 [5.20, 5.80]	12.2 [11.5, 13.2]	15.1 [12.0, 15.2]	16.2 [16.0, 21.9]	22.2 [21.8, 22.3]
Large beads: Size (mm)					
Mean (SD)	8.47 (0.468)	16.4 (7.35)	21.6 (12.0)	22.9 (12.4)	29.8 (17.8)
Median[Min, Max]	8.30 [8.10, 9.30]	16.4 [9.10, 23.8]	22.8 [9.10, 33.0]	26.5 [9.10, 33.0]	40.0 [9.20, 40.2]
Immersion fluid	PEG	Psyllium	Splenda		
Time when measured	24 h	24 h	24 h		
	(*N* = 3)	(*N* = 3)	(*N* = 3)		
Small beads: Size (mm)					
Mean (SD)	8.40 (1.61)	10.5 (1.47)	10.2 (0.513)		
Median[Min, Max]	8.20 [6.90, 10.1]	9.70 [9.60, 12.2]	10.1 [9.80, 10.8]		
Medium beads: Size (mm)					
Mean (SD)	16.8 (5.93)	25.0 (2.27)	24.3 (2.30)		
Median[Min, Max]	16.1 [11.3, 23.1]	24.3 [23.1, 27.5]	24.3 [22.0, 26.6]		
Large beads: Size (mm)					
Mean (SD)	16.5 (7.20)	28.4 (1.80)	31.4 (0.400)		
Median[Min, Max]	16.6 [9.20, 23.6]	28.4 [26.6, 30.2]	31.4 [31.0, 31.8]		

In summary, the results highlight that the expansion capacity of super absorbent polymer beads is influenced by both bead size and solution type. Small beads showed limited growth in acidic conditions but significant expansion in psyllium and saccharin solutions. Medium and large beads demonstrated remarkable expansion, particularly in tap water and simulated small intestine fluid. Among the treatment solutions, saccharin consistently facilitated the greatest bead growth, particularly for large beads. These findings emphasize the importance of solution composition in influencing the hydration and expansion dynamics of super absorbent polymers.

In all solutions, a statistically significant correlation was found between the dry size of the bead and the maximum diameter it could reach upon hydration regardless of the solution type. Multivariable Linear mixed modeling further quantified the effect of bead size, liquid type, and time on expansion. Medium beads showed a significant increase in size with a coefficient of 8.25 (95% CI: 4.38–12.13; *p* < 0.001) and large beads with a *β* of 11.87 (95% CI: 8.07–15.68; *p* < 0.001) compared to small beads. For liquid type, gastric acid significantly reduced expansion with a *β* of −5.16 (95% CI: −8.9 to −1.42; *p* < 0.001) and alkalotic solution with a *β* of −2.21 (95% CI: −5.94 to 1.53; *p* = 0.002) compared to water. Time also played a significant role, with the 24-h mark showing the largest increase in size with a *β* of 12.6 (95% CI: 10.09–15.12; *p* < 0.001). These results demonstrate a size-dependent and solution-specific impact on the expansion of water beads, with larger sizes expanding more significantly over time and certain solutions like gastric acid limiting expansion. The maximum diameter attained by the water beads varied significantly across the samples, ranging to 72 mm.Unsurprisingly, a statistically significant correlation was found between the dry size of the bead and the maximum diameter it could reach upon hydration.

**Figure 1:**
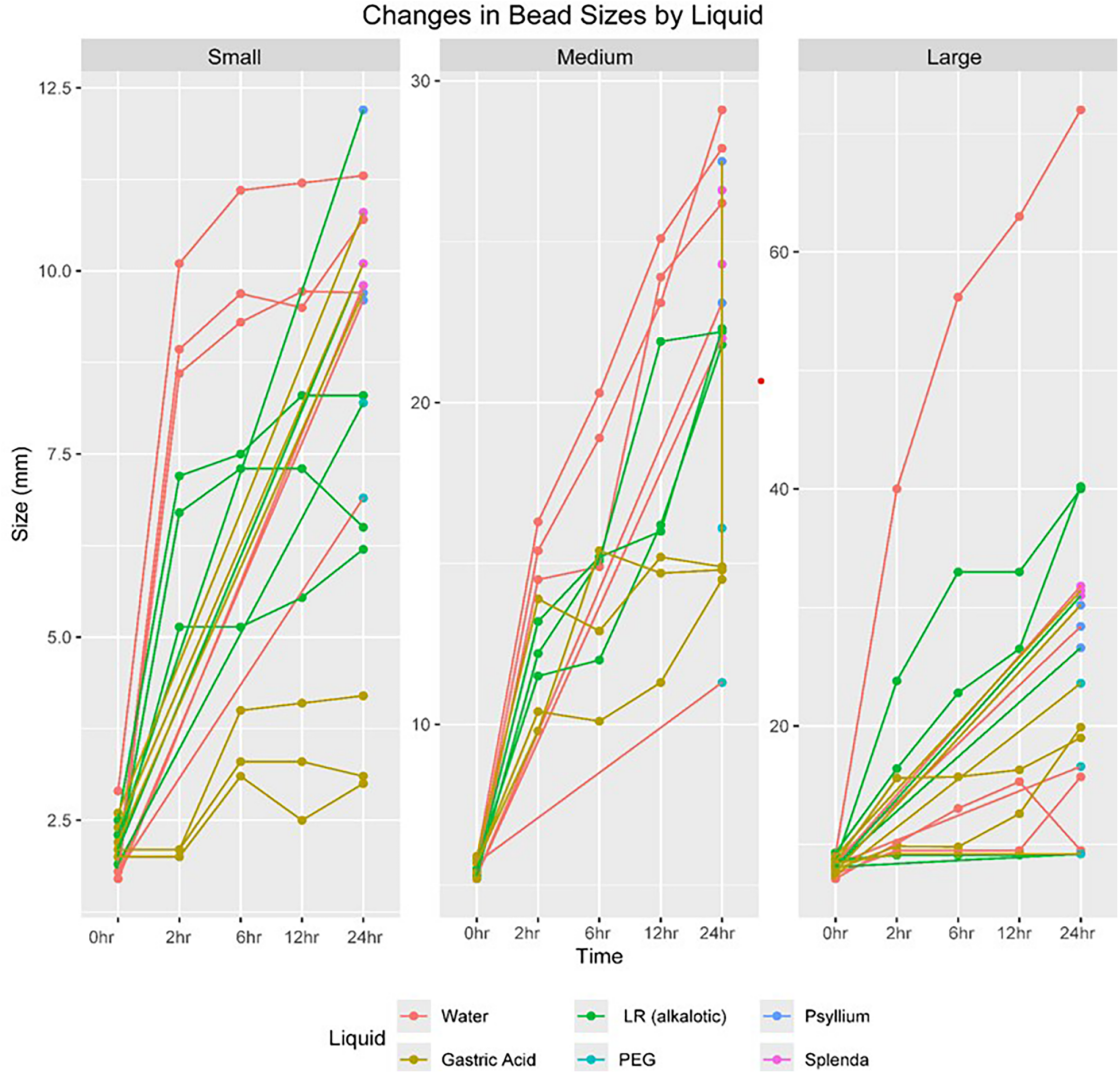
Graphical representation of the size dependent effect on maximum growth.

After 2 h of exposure to the initial control solutions, three water beads were randomly selected from each group and introduced to three unique treatment solutions: PEG 3350 (17 grams in 6 oz), fiber (3 grams in 6 oz), and hyperosmolar Splenda water (1 gram in 6 oz). Our observations showed that the water beads exhibited the least growth in the PEG 3350 solution. Specifically, the small beads expanded to a mean diameter of 8.4 mm (SD ± 1.61) the medium beads to 16.8 mm (SD: ±5.93), and the large beads to 16.5 mm (SD: ±7.2) in the 17-gram PEG 3350 solution. The detailed data of experiment 1 are outlined in Table 1 and Figure 1. This experiment provides important insights into the variability in water bead expansion across different sizes and solution types and emphasizes the predictive value of the bead's dry size.

### Experiment 2: analysis of bead characteristics on the expansion of SAP beads

In the second experiment, the expansion of water beads from six different brands—AINOLWAY, BABIYA, COSMOS, LEECHE, MAGIC LABS, and YIQUO—was compared over 72 h when submerged in water. The initial mean diameters of the beads varied significantly across brands, ranging from 2.05 to 7.41 mm, with a *p*-value <0.001, indicating a statistically significant difference.

**Figure 2:**
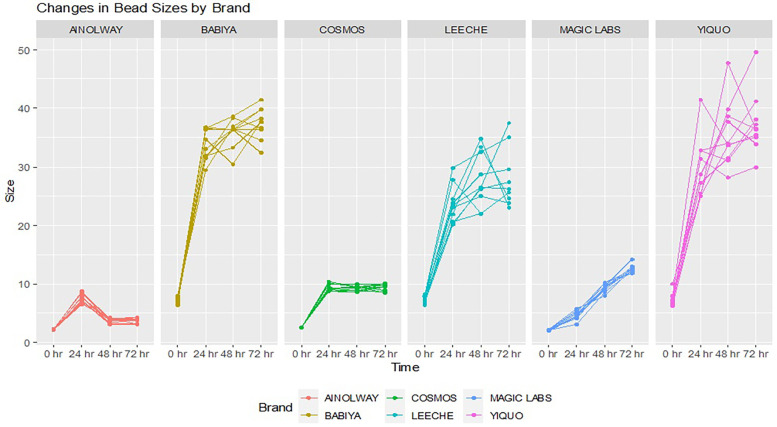
Graphical representation of the product related effect on maximum growth.

Over 24 h, the beads expanded, with BABIYA showing the most substantial growth (mean diameter 33.8 mm), and AINOLWAY the least (mean diameter 7.55 mm). This trend continued through 48 and 72 h, with BABIYA beads maintaining the largest size and AINOLWAY the smallest. The overall mean diameters at 48 and 72 h across all brands were 20.2 mm and 21.4 mm, respectively, both with *p*-values <0.001, affirming the significant differences in expansion among brands. Linear mixed model analysis provided a detailed examination of the time and brand effects on bead expansion. The time effect was significant at all measured points, with the greatest expansion observed at 72 h (*β* = 16.57; 95% CI: 14.63–18.5; *p* < 0.001). When looking at brand effects, BABIYA and YIQUO showed the largest size increases with *β* of 24.4 and 23.29, respectively (*p* < 0.001 for both). COSMOS and MAGIC LABS had smaller, yet significant, size increases (*β* of 3.39 and 2.89; *p* = 0.003 and *p* = 0.008, respectively). LEECHE also showed a substantial increase in size (*β* = 17.4; *p* < 0.001). The complete results are referenced in Table 2 and Figure 2 and demonstrate that both the time elapsed and the brand of water beads significantly affect the degree of expansion, with some brands showing markedly more substantial growth than others.

**Table 2:** Product-related effect on maximum growth.

SAP Brand	AINOLWAY	BABIYA	COSMOS	LEECHE	MAGIC LABS	YIQUO	Overall	*P*-value
	(*N* = 10)	(*N* = 10)	(*N* = 10)	(*N* = 10)	(*N* = 10)	(*N* = 10)	(*N* = 60)	
0 h								Kruskal–Wallis test
Mean (SD)	2.15 (0.0850)	7.25 (0.484)	2.54 (0.0474)	7.41 (0.567)	2.05 (0.0850)	7.36 (1.07)	4.79 (2.62)	<0.001
Median [Min, Max]	2.15 [2.00, 2.30]	7.25 [6.40, 7.90]	2.50 [2.50, 2.60]	7.60 [6.40, 8.20]	2.05 [1.90, 2.20]	7.15 [6.20, 10.0]	4.40 [1.90, 10.0]	
Median (IQR)	2.15 (2.10, 2.20)	7.25 (7.00, 7.60)	2.50 (2.50, 2.59)	7.60 (6.98, 7.78)	2.05 (2.00, 2.10)	7.15 (6.75, 7.68)	4.40 (2.20, 7.23)	
24 h								Kruskal–Wallis test
Mean (SD)	7.55 (0.805)	33.8 (2.68)	9.36 (0.560)	23.5 (3.20)	4.57 (0.767)	29.8 (5.06)	18.1 (11.8)	<0.001
Median [Min, Max]	7.40 [6.50, 8.70]	33.9 [29.4, 36.7]	9.20 [8.70, 10.4]	23.2 [20.1, 29.8]	4.45 [3.00, 5.80]	28.7 [24.9, 41.4]	15.3 [3.00, 41.4]	
Median (IQR)	7.40 (6.93, 8.25)	33.9 (31.8,36.5)	9.20 (9.03, 9.68)	23.2 (20.9,24.3)	4.45 (4.30, 5.05)	28.7 (25.9,32.4)	15.3 (7.55, 28.9)	
48 h								Kruskal–Wallis test
Mean (SD)	3.61 (0.428)	36.0 (2.41)	9.18 (0.487)	27.8 (4.53)	9.27 (0.695)	35.6 (5.59)	20.2 (13.7)	<0.001
Median [Min, Max]	3.75 [3.00, 4.20]	36.4 [30.5, 38.7]	9.30 [8.50, 10.0]	26.5 [22.0, 34.8]	9.35 [7.90, 10.2]	33.9 [28.1, 47.7]	16.1 [3.00, 47.7]	
Median (IQR)	3.75 (3.23, 3.88)	36.4 (36.3,36.8)	9.30 (8.80, 9.48)	26.5 (25.3,31.6)	9.35 (8.98, 9.55)	33.9 (32.0,38.5)	16.1 (8.80, 33.7)	
72 h								Kruskal–Wallis test
Mean (SD)	3.60 (0.462)	37.5 (2.67)	9.39 (0.606)	27.8 (4.86)	12.6 (0.680)	37.3 (5.21)	21.4 (13.9)	<0.001
Median [Min, Max]	3.75 [3.00, 4.20]	37.7 [32.4, 41.5]	9.45 [8.40, 10.1]	25.9 [23.0, 37.5]	12.5 [11.7, 14.2]	36.4 [29.9, 49.6]	18.6 [3.00, 49.6]	
Median (IQR)	3.75 (3.13, 3.88)	37.7 (36.5,39.4)	9.45 (8.95, 9.93)	25.9 (24.8,29.1)	12.5 (12.2,12.8)	36.4 (35.1,37.9)	18.6 (9.48, 35.6)	

### Experiment 3: analysis of PEG 3350 effect on the expansion of SAP beads

In Experiment 3, the diameter expansion of water beads was assessed at 24 and 48 h after soaking in various concentrations of polyethylene glycol (PEG) 3350. Beads were initially conditioned in simulated gastric acid followed by an alkaline solution before exposure to the PEG solutions.

At the 24-h mark, a significant expansion was observed across all PEG concentrations when compared to the baseline (Kruskal–Wallis test, *p* < 0.001). Beads in the 17 g of PEG in 3 oz of water solution had a mean diameter of 17.9 mm (SD: 1.89); median (IQR): 17.5 (17.1,17.8) and those in 17 g of PEG in 6 oz of water demonstrated a larger mean diameter of 21.8 mm (SD: 1.74); median (IQR): 22.1 (20.3,22.6). The smallest mean diameter of 10.4 mm (SD: 0.531) median (IQR): 10.4 (10.1,10.8) was found in the 51 g PEG in 3 oz of water group, while the 34 g in 6 oz solution resulted in a relatively larger mean diameter of 19.4 mm (SD 1.68); median (IQR): 18.9 (18.5,19.5).

At the 48-h interval, the increase in diameter persisted with statistical significance (Kruskal–Wallis test, *p* < 0.001). The mean diameter in the 17 g PEG in 6 oz water group was 23.6 mm (SD 1.91); median (IQR): 23.7 (21.8, 25.3), reflecting the continued swelling of beads. The 51 g PEG in 3 oz water group maintained the trend of smaller bead sizes with a mean diameter of 10.9 mm (SD 0.829); median (IQR): 10.9 (10.1, 11.6).

**Figure 3:**
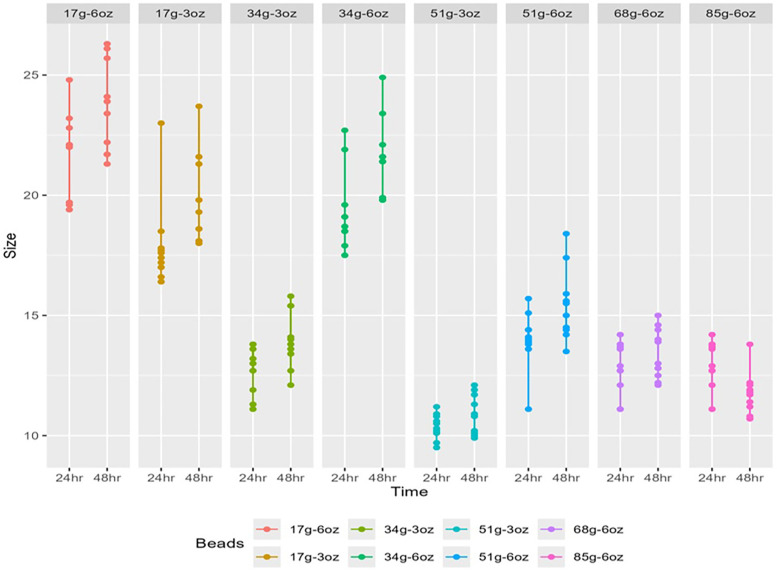
Graphical representation of the PEG 3350 related effect on maximum growth.

In the multivariable analysis, time within groups was a significant factor for diameter expansion. The 48-h time point was associated with an increased bead diameter [mean increase = 1.12, 95% CI (0.7, 1.54), *p* < 0.001] compared to at 24 h. In univariate analysis, time was not a significant factor at 24 h [mean increase = 1.1, 95% CI (−0.22, 2.5), *p* = 0.1], suggesting that other variables may contribute to diameter changes at this time point. Between-group comparisons showed that concentration of PEG was significantly associated with bead size. The largest decreases were observed in high concentrations of PEG with smaller volumes of water, indicating a decrease in bead size. Specifically, 51 g of PEG in 3 oz of water had a significant shrinkage effect [mean decrease of 12.07 mm, 95% CI (−13.02, −11.12), *p* < 0.001] compared to 17 g of PEG in 6 oz of water. The least shrinkage effect within the high concentration groups was observed with 34 g of PEG in 6 oz of water [mean decrease of 2.23 mm, 95% CI (−3.18, −1.29), *p* < 0.001] compared to 17 g of PEG in 6 oz of water. Complete data can be referenced in Figure 3, Tables 3, 4.

**Table 3:** PEG 3350 related effect on maximum growth.

Details of immersion fluid	17 g-3oz	34 g-3oz	51 g-3oz	Overall	*P*-value
	(*N* = 10)	(*N* = 10)	(*N* = 10)	(*N* = 80)	
24 h					Kruskal–Wallis test
Mean (SD)	17.9 (1.89)	12.7 (0.985)	10.4 (0.531)	15.2 (3.95)	<0.001
Median [Min, Max]	17.5 [16.4, 23.0]	12.9 [11.1, 13.8]	10.4 [9.50, 11.2]	13.8 [9.50, 24.8]	
Median (IQR)	17.5 (17.1,17.8)	12.9 (12.1,13.5)	10.4 (10.1,10.8)	13.8 (12.6,18.1)	
48 h					Kruskal–Wallis test
Mean (SD)	19.9 (1.82)	14.0 (1.20)	10.9 (0.829)	16.3 (4.68)	<0.001
Median [Min, Max]	19.6 [18.0, 23.7]	13.9 [12.1, 15.8]	10.9 [9.90, 12.1]	14.8 [9.90, 26.3]	
Median (IQR)	19.6 (18.6,20.9)	13.9 (13.5,15.1)	10.9 (10.1,11.6)	14.8 (12.2,20.3)	
Details of immersion fluid	17 g-6oz	34 g-6oz	51 g-6oz	68 g-6oz	85 g-6oz
	(*N* = 10)	(*N* = 10)	(*N* = 10)	(*N* = 10)	(*N* = 10)
24 h					
Mean (SD)	21.8 (1.74)	19.4 (1.68)	14.0 (1.20)	12.8 (1.09)	12.8 (1.09)
Median [Min, Max]	22.1 [19.4, 24.8]	18.9 [17.5, 22.7]	14.1 [11.1, 15.7]	12.8 [11.1, 14.2]	12.8 [11.1, 14.2]
Median (IQR)	22.1 (20.3,22.6)	18.9 (18.5,19.5)	14.1 (13.8,14.4)	12.8 (12.3,13.7)	12.8 (12.3,13.7)
48 h					
Mean (SD)	23.6 (1.91)	21.6 (1.63)	15.4 (1.50)	13.5 (1.06)	11.8 (0.881)
Median [Min, Max]	23.7 [21.3, 26.3]	21.5 [19.8, 24.9]	15.3 [13.5, 18.4]	13.5 [12.1, 15.0]	11.8 [10.7, 13.8]
Median (IQR)	23.7 (21.8,25.3)	21.5 (20.3,22.0)	15.3 (14.4,15.8)	13.5 (12.6,14.3)	11.8 (11.3,12.1)

**Table 4:** Linear mixed model using time, size type and type of liquid as predictors.

Experiment 1			Experiment 2			Experiment 3		
Characteristic	*β* (95% CI)	*p*-value	Characteristic	*β* (95% CI)	*p*-value		Multivariable	
Time (Within group)			Time (Within group)			Characteristic	*β* (95% CI)	*p*-value
0 h	Ref		0 h	Ref		Time (Within Group)		
2 h	6.02 (3.5–8.53)	0.001	24 h	13.31 (11.38–15.25)	<0.001	24 h	Ref	
6 h	8.24 (5.72–10.75)	<0.001	48 h	15.44 (13.5–17.37)	<0.001	48 h	1.12 (0.7,1.54)	<0.001
12 h	10.08 (7.57–12.6)	<0.001	72 h	16.57 (14.63–18.5)	<0.001			
24 h	12.6 (10.09–15.12)	<0.001				Dilutant (Between Group)		
						3oz	Ref	
Type (Between group)			Brand (Between group)			6oz	5 (4.36, 5.64)	<0.001
Small	Ref		AINOLWAY	Ref				
Medium	8.25 (4.38–12.13)	<0.001	BABIYA	24.4 (22.03–26.77)	<0.001	Grams of PEG (Between Group)		
Large	11.87 (8.07–15.68)	<0.001	COSMOS	3.39 (1.02–5.76)	0.003	17 g	Ref	
			LEECHE	17.4 (15.03–19.77)	<0.001	34 g	−3.88 (−4.66, −3.1)	<0.001
Liquid (Between group)			MAGIC LABS	2.89 (0.52–5.26)	0.008	51 g	−8.12 (−8.9, −7.34)	<0.001
Water	Ref		YIQUO	23.29 (20.92–25.66)	<0.001	68 g	−10.18 (−11.19, −9.17)	<0.001
Gastric Acid	−5.16 (−8.9 to 1.42)	0.003				85 g	−11.03 (−12.03, −10.02)	<0.001
LR (alkalotic)	−2.21 (−5.94 to 1.53)	0.124						
PEG	−5.93 (−11.00 to 0.86)	0.011						
Psyllium	1.07 (−4.00 to 6.14)	0.339						
Splenda	1.49 (−3.58 to 6.56)	0.282						

## Discussion

Our findings demonstrate a potential risk of obstruction in patients who ingest SAP beads larger than 3 mm in dry size, which can expand to sizes greater than 25 mm. This contrasts with previous studies that did not identify SAP beads as a risk, since they did not observe expansion to sizes posing an obstruction risk (6–8). However, these studies did not consider important characteristics that may influence the expansion to larger sizes, which can lead to serious consequences, including fatalities as reported in previous case studies (2). These studies did not account for the dry size of the water beads, which is a crucial determinant of their expansion potential. Our research specifically addresses this gap by thoroughly investigating how beads with different initial dry sizes respond in various liquid environments, thereby providing a clearer understanding of which beads pose the greatest risk. Additionally, prior studies did not consider the concentration of polyethylene glycol (PEG) 3350, commonly known as MiraLAX, which our study has shown can effectively reduce bead size. By exploring the impact of varying concentrations of MiraLAX, we provide valuable insights into potential therapeutic strategies for managing SAP bead ingestions. Moreover, previous research often lacked detailed information regarding the number and types of beads studied, leading to inconsistencies and limitations in the applicability of their findings. Our study addresses these omissions by clearly specifying the types and quantities of beads examined, along with their brand differences, which allows for a more comprehensive and reliable assessment of the risks associated with SAP bead ingestions.

Interestingly, not all SAP beads pose the same level of risk. This underscores the critical need for effective management and screening strategies, as current methods to mitigate obstruction risks are either unstructured or invasive. The *in vitro* data from this study helps identify the potential for size expansion associated with the dry size of the bead, the type of bead, and the different solutions that the SAP beads are exposed to. Notably, the larger the dry bead size the greater the potential for expansion to a size that could lead to obstruction. The beads appear to have the most rapid growth in the first 24 h, with the large SAP beads reaching a size >25 mm as early as 6 h. The beads did not exceed >20 mm in the gastric acid even at 24 h. This may suggest the reduced expansion of SAP beads in the stomach and that the SAPs will pass through the pylorus. This could also mean that antacids, histamine inhibitors, and proton pump inhibitors may influence the size of the SAP beads *in vivo* as well. *in vitro* data does not take into account the gastric motility and other fluids that may influence pH and osmolality such as refluxed bile, mucus and retained food/liquids. Polyethylene Glycol 3350 (PEG 3350) had the most significant impact on the potential growth of the SAP beads *in vitro*. In cases of SAP ingestion, PEG 3350 may be used as a potential treatment for patients, such as in other foreign body ingestions, provided they are on parenteral fluids and are closely monitored in a hospital setting (9). Even modest doses of PEG 3350 (17 grams in 6oz) led to a statistically significant limitation in the maximum expansion of SAP beads. As shown in the *in vitro* data, reducing water in the PEG 3350 solution results in an increase in the mass concentration that can enhance the osmotic effect in the gastrointestinal tract due to the higher number of PEG 3350 molecules retaining water. The more concentrated the greater the effect of PEG 3350. These findings have implications for the use of PEG 3350 in applications that require control over the expansion of hydrogel materials.

**Figure 4:**
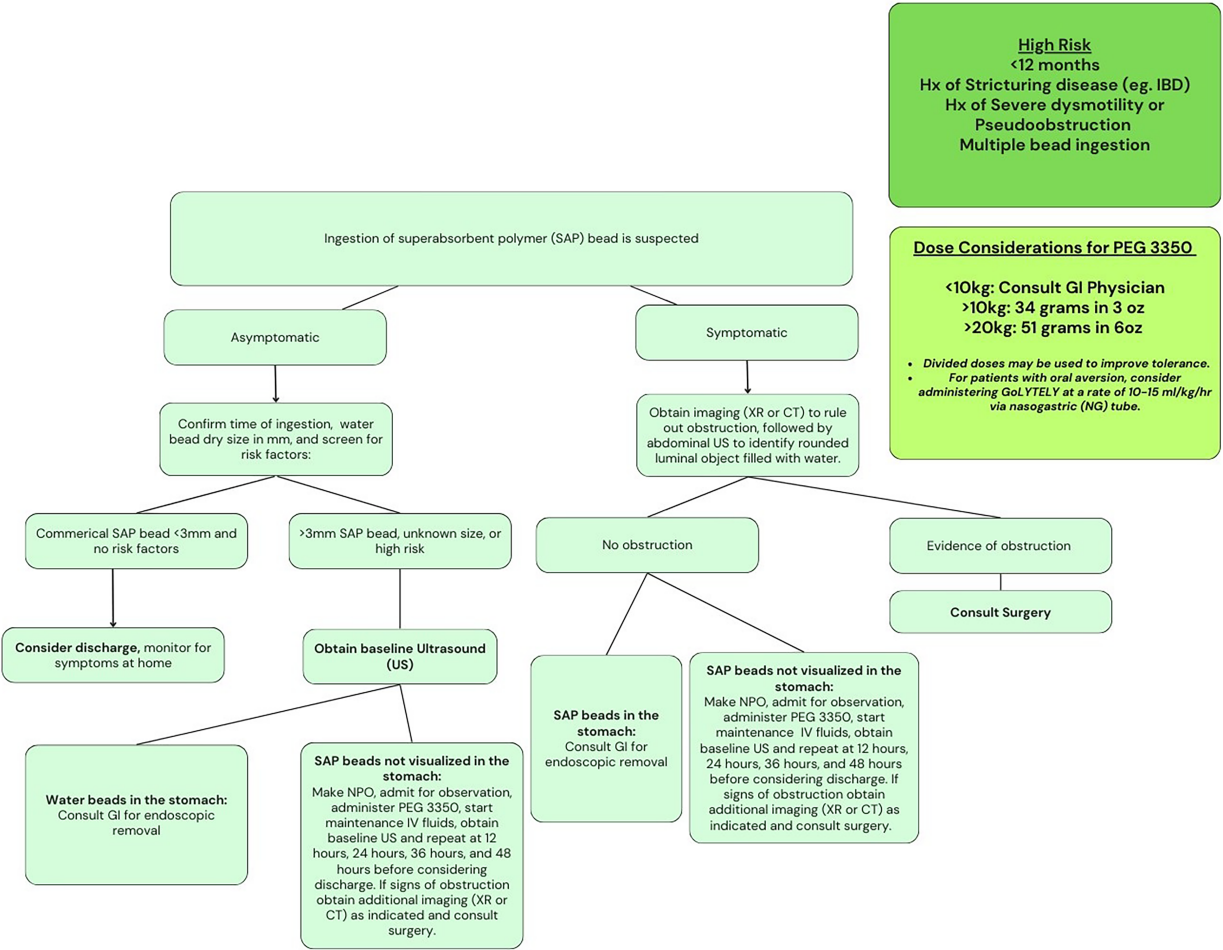
Proposed algorithm.

A proposed algorithm is included in Figure 4 based on the data gathered from this study and previous studies. This algorithm could serve as a potential guideline to help emergency department physicians and hospitalists develop an effective approach to patients who present after ingestion of SAP beads. It is crucial to obtain a history detailing the type of bead, when the bead was ingested, and if any significant risk factors pose a greater risk for obstruction. Garnering this information can help determine whether the patient needs hospitalization, imaging, or invasive intervention. x-ray imaging can be helpful in identifying obstructions, but are unlikely to detect SAP beads that are radiolucent. Ultrasound may be a more effective imaging technique for detecting water beads that have expanded without exposing patients to significant radiation. This was shown in a previous case series that looked at different imaging studies used to identify SAP beads (10). The recommended dosing for PEG 3350 is based on NAPGHAN guidelines and other studies that suggest safe doses as high as 2 g/kg (11, 12). Higher concentrations may increase the risk of dehydration, thus we recommend parenteral fluids. Regarding the safety of using more osmolar solutions than standard recommendations, this is not dissimilar to using GoLYTLEY which has a significantly higher osmolality than the both standard doses of PEG 3350 and the concentrated maximum doses PEG 3350 recommended. 34 grams in 3oz is approximately 114.4 mOsm/L and 51 in 6oz is approximately 85.8 mOsm/L compared to 300 mOsm/L with GoLYTELY.

### Limitations

Our study was conducted in an *in vitro* setting under controlled laboratory conditions, which inherently differ from the dynamic and variable physiological environment encountered in pediatric patients. Unlike real-world clinical scenarios, *in vitro* experiments do not account for the continuous gastric motility, secretion patterns, and the complex interplay of various endogenous fluids that may influence pH and osmolality. These include refluxed bile, mucus, and residual food or liquid content within the stomach, all of which can impact the dissolution, interaction, and potential effects of ingested materials. Additionally, the chemical properties of the different water beads are poorly defined, as there are no safety data sheets available. The properties of these beads are limited to the understanding that the gel is based on sodium polyacrylate. A more comprehensive understanding of other variables related to the properties of the beads could further help stratify risk.

Future studies should aim to simulate more accurately the *in vivo* environment by incorporating dynamic models that mimic gastric and intestinal conditions, such as immersing SAP beads in gastric acid followed by exposure to intestinal juices and studying this independently. Efforts to simulate peristalsis and the influences of other intestinal fluids, potentially through the use of a porcine intestine model, could provide a more comprehensive understanding of the behavior of SAP beads within the gastrointestinal tract. This approach, combined with a deeper investigation into the chemical properties of the beads, would help to bridge the gap between *in vitro* findings and their real-world clinical implications.

## Conclusions

This study highlights the significant expansion potential of super absorbent polymer (SAP) water beads in various liquid environments, underscoring the associated risks in pediatric ingestions. Our findings indicate that bead size and the composition of the surrounding fluid are critical factors influencing expansion. Beads with a dry size greater than 3 mm demonstrated substantial growth, particularly in water, posing a significant risk as they can expand to obstructive diameters exceeding 25 mm within a short timeframe. In contrast, beads smaller than 3 mm pose minimal risk. Gastric acid may limit expansion, whereas alkaline solutions seem to allow continued growth, presumably increasing the risk of intestinal obstruction post-gastric emptying. Differences in expansion between brands suggest that variations in the chemical properties of SAP gels used by manufacturers may also contribute to these differences. Lastly, our data suggest that higher concentrations of polyethylene glycol (PEG) 3350 can effectively reduce bead size, presenting a potential therapeutic strategy when patients are adequately monitored in a hospital setting.

The rapid and significant expansion of larger beads, coupled with the rise in ingestion cases, underscores the need for early identification and intervention to mitigate ingestion hazards. Given the lack of current guidance, further research is necessary to develop a safe and standardized approach to SAP ingestions in a clinical setting. Importantly, the *in vitro* data provided by this study could be crucial in better understanding the risk of SAP ingestions, ultimately guiding the development of safer, more efficient, and less invasive management strategies.

## Data Availability

The raw data supporting the conclusions of this article will be made available by the authors, without undue reservation.
